# Alhagi honey polysaccharide ameliorates carbon tetrachloride-induced liver fibrosis via modulating the gut microbiota-liver metabolism axis

**DOI:** 10.3389/fnut.2026.1862546

**Published:** 2026-05-28

**Authors:** Jianzhong Song, Xin Zhao, Lingling Cao, Yingying Chen, Xingchen Zhou, Jinfa Li, Peijin You, Jing Zhou, Zhiyuan Lv, Xiumin Ma, Junmin Chang

**Affiliations:** 1 Postdoctoral Research Workstation of Tumor Hospital affiliated to Xinjiang Medical University, Urumqi, China; 2 Department of Pharmacy, Tumor Hospital Affiliated to Xinjiang Medical University, Urumqi, China; 3 Department of Pharmacy, The First Affiliated Hospital of Xinjiang Medical University, Urumqi, China; 4 Xinjiang Cardiovascular and Cerebrovascular Disease Hospital Laboratory Department, Urumqi, Xinjiang, China; 5 Department of Pharmacy, Baiyin Hospital of Integrated Traditional Chinese and Western Medicine, Gansu, China; 6 Division of (Bio) Pharmaceutics, Institute of Zhejiang University-Quzhou, Quzhou, China; 7 College of Chemical and Biological Engineering, Zhejiang University, Hangzhou, China; 8 College of Pharmacy, Xinjiang Medical University, Urumqi, China; 9 Xinjiang Huachun Institute of Biomedical Research, Urumqi, China; 10 State Key Laboratory of Pathogenesis, Prevention and Treatment of High Incidence Diseases in Central Asia, Clinical Laboratory Center, Tumor Hospital Affiliated to Xinjiang Medical University, Urumqi, Xinjiang, China; 11 Xinjiang Key Laboratory of Natural Medicines Active Components and Drug Release Technology, Urumqi, China

**Keywords:** Alhagi honey polysaccharide, gut microbiota-liver metabolism axis, intestinal barrier, liver fibrosis, oxidative stress

## Abstract

**Background:**

Liver fibrosis (LF) is a progressive injury that frequently advances to cirrhosis and hepatocellular carcinoma, yet effective intervention strategies remain limited. Alhagi honey polysaccharide (AHPN), a bioactive macromolecule with a molecular weight of 9.35 × 10ł Da primarily composed of mannose (29.384%), glucose (41.804%), and galactose (28.810%), represents a promising candidate for antifibrotic therapy.

**Methods:**

This study established a carbon tetrachloride (CCl_4_)-induced liver fibrosis mouse model to evaluate AHPN’s intervention effect. Liver injury, collagen deposition, and fibrosis progression were assessed histologically and biochemically. The underlying mechanisms were explored through analysis of the TGF-β1/Smad3 signaling pathway, hepatic stellate cell activation, Nrf2/HO-1 antioxidant pathway, inflammatory cytokine profiles, and fibrosis marker expression. Additionally, 16S rDNA sequencing and metabolomics were employed to investigate AHPN’s regulatory effects on gut microbiota composition and hepatic metabolism.

**Results:**

AHPN administration significantly attenuated hepatic injury, reduced collagen deposition, and suppressed fibrosis progression by inhibiting TGF-β1/Smad3 signaling and hepatic stellate cell activation, accompanied by restored liver function. Mechanistically, AHPN activated the Nrf2/HO-1 pathway to alleviate oxidative stress, decreased serum levels of pro-inflammatory cytokines (IL-6, IL-1β, TNF-α), and downregulated fibrosis-related markers (α-SMA, collagen I, LN, PCIII, IV-C). Notably, 16S rDNA sequencing revealed that AHPN enriched beneficial gut bacteria including *Bacteroidaceae*, *Lactobacillaceae*, and *Marinifilaceae* while restoring intestinal tight junction proteins (Occludin, ZO-1). Metabolomics analysis further demonstrated that AHPN improved hepatic metabolic disorders.

**Conclusion:**

These findings indicate that AHPN ameliorates CCl_4_-induced liver fibrosis through a multi-targeted mechanism involving suppression of TGF-β1/Smad3 signaling, activation of the Nrf2/HO-1 antioxidant pathway, reduction of inflammatory responses, and crucially, modulation of the gut microbiota-liver metabolism axis. These findings underscore the potential of dietary polysaccharides in modulating the gut microbiota-liver metabolism axis and provide a basis for developing nutritional strategies against liver fibrosis.

## 1 Introduction

Liver fibrosis represents a pathological wound-healing response to sustained hepatic injury, characterized by excessive extracellular matrix (ECM) deposition and progressive alteration of liver architecture (1, 2). Persistent insults from parasitic infections, alcohol overconsumption, drug toxicity, metabolic syndrome, and cholestasis frequently precipitate this condition (3). Although liver fibrosis is reversible in early stages (4, 5), delayed intervention leads to irreversible cirrhosis and hepatocellular carcinoma (6). Currently, no approved nutritional or pharmacological intervention specifically targets fibrosis reversal, underscoring the need for novel strategies that modulate metabolic and microbial pathways underlying disease progression (7).

The liver functions as the central metabolic organ, and chronic liver diseases invariably involve metabolic perturbations including oxidative stress, lipid peroxidation, and mitochondrial dysfunction (8, 9). Carbon tetrachloride (CCl_4_), a classic hepatotoxicant, induces experimental liver injury through free radical-mediated lipid peroxidation and antioxidant depletion (10, 11), thereby recapitulating key metabolic disturbances observed in human steatohepatitis and alcohol-induced liver disease. The resulting oxidative stress triggers inflammatory cascades that amplify liver damage (12, 13), establishing CCl_4_ as a widely accepted model for investigating metabolic interventions in liver pathology.

The gut microbiota-liver metabolism axis constitutes a critical bidirectional communication system linking intestinal microbial ecology with hepatic metabolic homeostasis (14). Gut-derived metabolitesd mestasis itical b-chain fatty acids, bile acid derivatives, and amino acid metabolitesfatty acids, bile acid derivatives, and amino acid melinking intestinal microbi(15). Dietary polysaccharides, which resist upper gastrointestinal digestion and reach the colon intact, serve as selective substrates for specific microbial taxa, thereby reshaping community structure and function (16). Integrating 16S rDNA sequencing with untargeted metabolomics provides a powerful approach to dissect how dietary components modulate this axis in liver disease pathogenesis and intervention.

*Alhagi sparsifolia* Shap., a drought-resistant shrub distributed across saline-alkaline regions of Northwest China and Central Asia, produces Alhagi honeyity structure and function ervource consisting of solid sugar granules exuded from leaf surfaces (17). In traditional Uyghur medicine, this honey has been applied for hepatobiliary disorders, dysentery, and diarrhea (18). Our previous studies identified the primary bioactive constituent as a polysaccharide, AHPN (molecular weight: 9.35 × 10^3^ Da), composed of mannose, glucose, and galactose. This polymer demonstrated anti-inflammatory, antioxidant, and intestinal barrier-protective properties in an acute alcoholic liver injury model (19). Given the established role of gut dysbiosis, intestinal barrier dysfunction, and hepatic metabolic disorders in driving fibrogenesis, we speculate that AHPN may exert protective effects against liver fibrosis primarily through modulation of the gut microbiota-liver metabolism axis. The present study was designed to evaluate whether AHPN attenuates CCl_4_-induced hepatic oxidative damage and inflammatory responses, and to elucidate its regulatory effects on fibrotic markers including α-smooth muscle actin (α-SMA), collagen type I, and TGF-β1/Smad3 signaling. Using 16S rDNA sequencing and LC-MS-based metabolomics, we further investigated how AHPN remodels intestinal microbial communities and hepatic metabolic profiles to achieve antifibrotic effects.

## 2 Materials and methods

### 2.1 Chemical substances and reagents

The materials for this study were procured from various commercial sources. Alhagi honey (Batch No. 20230516) was obtained from Xinjiang Medison Pharmaceutical Co., Ltd. Key reagents included olive oil (Shanghai Macklin Biochemical Technology Co., Ltd.). Oxidative stress markers were assessed using kits from different suppliers: superoxide dismutase (SOD) and glutathione peroxidase (GSH-Px) kits (Nanjing Jiancheng, Nos. A001-1 and A005), and a malondialdehyde (MDA) kit (Servicebio, No. G4300-96T). Serum biochemical parameters (ALT, AST, ALb) were measured with kits from Rayto (catalog Nos. S03030, S03040, S03043). A panel of enzyme-linked immunosorbent assay (ELISA) kits was used to quantify mouse cytokines (IL-6, TNF-α, IL-1β) and fibrosis markers (PcIII, LN, ColIV). For immunoblotting, primary antibodies against HO-1, Nrf2, TGF-β1, α-SMA, collagen I, Smad3, and p-Smad3, along with the GAPDH loading control, were sourced from Abcam (Cambridge, United Kingdom). Antibodies specific to the tight junction proteins ZO-1 and occludin were purchased from Servicebio (China). Other chemical reagents shall be of analytical grade.

### 2.2 Preparation of polysaccharide fractions from Alhagi honey

Samples of Alhagi honey were collected in Xinjiang, China, in March 2012. Authentication was performed by Dr. Junmin Chang, with voucher specimens deposited at the Plant Herbarium of Xinjiang Medical University (Accession No. 201203). The dried Alhagi honey was first defatted with petroleum ether and subsequently treated with 95% ethanol to remove alcohol-soluble components. Crude polysaccharides were obtained by extracting the residue with hot distilled water, concentrating the extract, and precipitating it with ethanol (80% final concentration). A homogeneous polysaccharide fraction, designated AHPN, was obtained from the crude polysaccharides by sequential purification with DEAE-650M anion-exchange and Sephacryl S-300 gel filtration chromatography, after prior decolorization using XDA-1 macroporous resin (19).

### 2.3 Apparent molecular weight and thermal characterization of AHPN

The molecular weight of AHPN was determined by high-performance liquid chromatography coupled with size-exclusion chromatography (HPLC-SEC) using a TSK-gel G3000PWXL column and refractive index detection. Dextran standards (5–2,000 kDa) were used to construct a calibration curve of retention time versus log molecular weight. AHPN (2 mg) was dissolved in 2 mL deionized water and analyzed under the same conditions. Thermal analysis was performed with a thermogravimetric analyzer (30–700–°C, 10°C/min), providing TG, DTG, and DSC data.

### 2.4 Microstructural and chemical analysis via SEM and FT-IR

The surface morphology of AHPN was characterized by scanning electron microscopy (SEM) using a SUPRA 55VP instrument (ZEISS, Germany). Dried AHPN powder (1 mg) was coated with a thin gold film, mounted on an aluminum stub, and observed at magnifications of × 500 and × 3,000. For Fourier-transform infrared (FT-IR) analysis, each sample (2 mg) was mixed with KBr powder and compressed into a thin disk. Spectral data were then collected using an IRPrestige-21 spectrometer (SHIMADZU, Japan) across a frequency range of 4,000–400 cm^−1^.

### 2.5 Monosaccharide composition analysis by GC

Briefly, 6 mg of the polysaccharide was hydrolyzed with 2 M trifluoroacetic acid (TFA) at 110 °C for 4 h. After hydrolysis, residual TFA was removed by repeated co-evaporation with methanol under reduced pressure. The resulting hydrolysate was then converted into alditol acetates by sequential derivatization: first with hydroxylamine hydrochloride (8 mg) and pyridine (0.5 mL), followed by acetylation with acetic anhydride for 10 min. The derivatives were subsequently dried under a stream of nitrogen, re-dissolved in chloroform, and subsequently analyzed by gas chromatography (GC).

### 2.6 Animals and treatments

**FIGURE 1:**
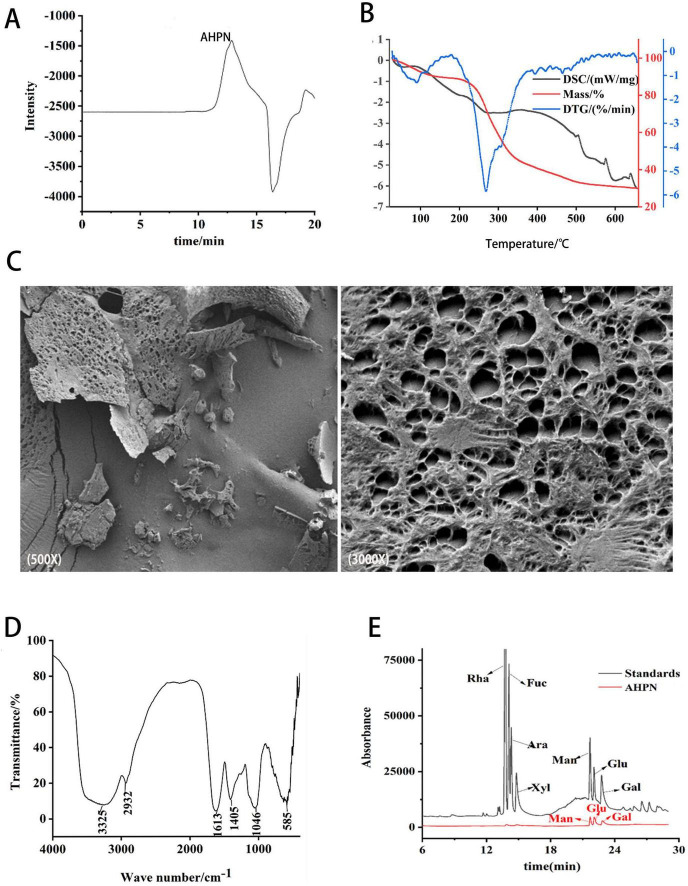
Characterization of AHPN structure. **(A)** The HPLC-SEC chromatogram of AHPN. **(B)** TG, DTG, and DSC curve for AHPN. **(C)** Scanning electron micrographs of AHPN (×500 and ×3,000). **(D)** FT-IR spectrum of AHPN. **(E)** Gas chromatograms illustrating monosaccharide composition: standards and AHPN.

Male C57BL/6 mice weighing 18.0–22.0 g were acclimatized for one week under standard laboratory conditions (23 ± 2°C, 50 ± 20% humidity, 12-h light/dark cycle) with free access to food and water. All animal protocols were approved by the Animal Experiment Ethics Committee of Xinjiang Medical University (License No. IACUC-20231010-20) and conformed to the NIH guidelines. After acclimatization, the animals were randomly assigned to five groups (*n* = 6 each): normal control (CTL), model group (Model), low-dose AHPN (LAHPN, 200 mg/kg), high-dose AHPN (HAHPN, 400 mg/kg), and silybin positive group (Positive, 150 mg/kg). Liver fibrosis was induced by intraperitoneal injection of 10% CCl_4_ in olive oil (2 mL/kg) two times weekly for 4 weeks; CTL mice received olive oil only. AHPN, dissolved in 0.5% sodium carboxymethyl cellulose (CMC-Na), was administered daily by oral gavage at 10 mL/kg at the indicated doses. The CTL group received an equal volume of 0.5% CMC-Na solution to control for any potential vehicle effects. The complete experimental timeline is depicted in Figure 1A. At the end of the intervention, 24 h after the final administration, mice were anesthetized with isoflurane (induction: 3–4%; maintenance: 1.5–2% in 100% O_2_, flow rate 1 L/min) via a nose cone. Once the depth of anesthesia was confirmed by the absence of a pedal withdrawal reflex, blood was collected from the orbital sinus. After blood collection, the mice were euthanized by cervical dislocation under deep isoflurane anesthesia. Liver tissues were immediately excised, rinsed with ice-cold saline, and weighed to calculate the liver index (liver weight/final body weight). For further analysis, liver specimens were processed accordingly: one portion was homogenized in saline for oxidative stress marker assessment; another was fixed in 10% formalin for histopathological examination.

### 2.7 Histopathological analysis

Liver and colon tissues harvested for histopathological examination were fixed in 4% paraformaldehyde for 48 h, then conventionally processed through graded ethanol, embedded in paraffin, and sectioned at 5 μm thickness. Hematoxylin and eosin (H&E) staining was employed for morphological assessment, while Masson’s trichrome and Sirius red staining—performed under analogous conditions—were used to evaluate fibrotic changes. Stained sections were examined under an Eclipse Ci-L upright light microscope (Nikon, Japan) and imaged for documentation. Quantification of collagen deposition was performed using Image-Pro Plus 6.0.

### 2.8 Biochemical analysis

After collection, blood samples were allowed to stand undisturbed for 2 h. Serum was isolated by centrifugation at 3,000 rpm for 15 min, and the supernatant was harvested for biochemical analysis. Levels of alanine aminotransferase (ALT), aspartate aminotransferase (AST), albumin (ALb), laminin (LN), procollagen type III (PIIINP), and collagen type IV (Col-IV) were determined using commercial kits, following the manufacturer’s instructions.

### 2.9 Measurement of oxidative stress indicators

SOD and GSH-Px activities, along with MDA levels, were determined in liver tissue supernatants prepared according to the protocol described in section 2.6, using commercially available kits obtained from the Nanjing Jiancheng Bioengineering Institute (Nanjing, China).

### 2.10 Detection of serum inflammatory cytokines and LPS

The levels of pro-inflammatory cytokines IL-1β, IL-6, TNF-α, and LPS in serum were quantified using commercial ELISA kits, allowing for assessment of the anti-inflammatory properties of AHPN.

### 2.11 Fluorescence microscopic detection of liver reactive oxygen species (ROS)

Liver ROS levels were evaluated by staining 10-μm cryosections with 5 μM dihydroethidium (DHE) at 37°C for 30 min. Upon oxidation by intracellular reactive oxygen species, DHE intercalates with DNA to yield nuclear red fluorescence. For each sample, images were captured from three randomly selected fields of view using a fluorescence microscope, and the mean fluorescence density (MFD), defined as the integrated fluorescence intensity per unit area, was quantified according to a previously described protocol (20).

### 2.12 Immunohistochemistry

Formalin-fixed, paraffin-embedded liver sections were routinely dewaxed after sectioning. Endogenous peroxidase activity was eliminated by 10-min treatment with 0.3% H_2_O_2_, followed by blocking of nonspecific binding sites with 5% bovine serum albumin (BSA) for 60 min. The sections were then incubated overnight at 4°C with primary antibodies directed against α-SMA and COL1A1. After washing, the sections were incubated with biotin-conjugated goat anti-mouse IgG for 1 h at room temperature, then with streptavidin-biotin complex (SABC; Boster, United States) for 30 min. Quantitative analysis of protein expression was performed by calculating the ratio of the positively stained area to the total field area using Image J software.

### 2.13 Immunofluorescence analysis

After fixation, the tissue sections underwent permeabilization and were subsequently treated with a blocking solution composed of 5% BSA for 60 min to reduce background staining. Following overnight incubation at 4°C with primary antibodies targeting Nrf2, HO-1, ZO-1, and Occludin, the sections were probed with fluorophore-conjugated secondary antibodies for 1 h. Nuclei were counterstained with DAPI for 10 min. Immunofluorescent images were subsequently processed with Image J software to determine the proportion of positively stained areas.

### 2.14 Western blotting

Extract liver tissue protein. Equal amounts of protein were resolved by SDS-PAGE, electro transferred to PVDF membranes, and blocked with 5% skimmed milk for 1 h at room temperature. The membranes were then incubated overnight at 4°C with gentle shaking using primary antibodies targeting Nrf2, HO-1, TGF-β1, Smad3, α-SMA, and Collagen I. After thorough washing, HRP-conjugated goat anti-rabbit secondary antibodies were applied for 2 h at ambient temperature. Protein bands were visualized and their intensities quantified using a Tanon 5200 chemiluminescence system.

### 2.15 Gut microbiota profiling via 16S rDNA sequencing

Using the cetyltrimethylammonium bromide (CTAB) method, total microbial DNA was isolated from fecal specimens collected from the CTL, Model, and HAHPN high-dose (400 mg/kg) groups, after which DNA purity and concentration were measured with a Nanodrop spectrophotometer. PCR amplification of the bacterial 16S rRNA gene’s V3–V4 hypervariable region (∼250 bp) was subsequently carried out using barcoded primers 338F (5′-Barcode-ACTCCTACGGGAGGCAGCA-3′) and 806R (5′-GGACTACHVGGGTWTCTAAT-3′). After quantification, amplified products were pooled at equimolar ratios. Libraries were quality-controlled, quantitated, and sequenced on an Illumina high-throughput platform. Following data consolidation, Uparse was employed to cluster operational taxonomic units (OTUs), after which representative sequences were mapped to the Greengenes database to obtain taxonomic classifications. Based on the annotation results, Linear Discriminant Analysis Effect Size (LEfSe) was further employed to identify significantly differential marker species (dominant microbiota) between groups. Alpha diversity and Beta diversity analyses were simultaneously conducted to assess differences in microbial community structure.

### 2.16 Antibiotic cocktail treatment

After 1 week of acclimation, C57BL/6 mice were divided into four groups (*n* = 6/group). Except controls, all received intraperitoneal CCl_4_ (10% in olive oil, 2 mL/kg) twice weekly for 4 weeks. The ABX and ABX + AHPN groups received antibiotics (vancomycin, ampicillin, neomycin, metronidazole) in drinking water. The ABX + AHPN and AHPN groups were orally given AHPN (400 mg/kg/day). After the final injection, mice were fasted for 24 hours, then anesthetized for sample collection.

### 2.17 Untargeted metabolomics of liver tissue

Extraction of liver samples (25 mg) was carried out in 500 μL of solvent by three repeated cycles of grinding (35 Hz, 4 min) and ice-water bath ultrasonication (5 min). After incubation at 40°C for 1 h, the mixture was centrifuged at 12,000 rpm for 20 min at 4°C. Chromatographic separation was achieved on a Vanquish UPLC system. The mobile phase, composed of 0.1% formic acid in water (A) and acetonitrile (B), was delivered at 0.3 mL/min with a 1.0 μL injection volume. Mass spectrometric analysis was conducted using electrospray ionization (ESI) in both positive and negative modes, with data acquired across an m/z range of 50–1,500 at a resolution of 70,000.

The acquired LC-MS datasets were subjected to Compound Discoverer 3.0 processing for feature extraction, chromatographic alignment, and relative quantification. Multivariate statistical analysis was conducted using SIMCA 14.1. Principal Component Analysis (PCA) was used to evaluate group separation, with model validity assessed via R^2^ and Q^2^. Metabolites with Variable Importance in Projection (VIP) > 1 and two-tailed *t*-test *p* < 0.05 were considered significantly changed. Quantitative data are expressed as mean ± standard deviation of normalized peak areas, with significance assessed by *t*-test (*p* < 0.05). Z-score normalized intensities of differential metabolites were visualized in a heatmap using the R package Pheatmap. Pearson correlation coefficients among these metabolites were calculated using cor() and cor.mtest() (*p* < 0.05), and visualized with corrplot. Ultimately, pathway enrichment analysis was conducted using the Kyoto Encyclopedia of Genes and Genomes (KEGG) database. A pathway was defined as significantly enriched when the proportion of differential metabolites was greater than background and *p* < 0.05.

### 2.18 Statistical analysis

Statistical analyses were performed using GraphPad Prism 8.0, with all data expressed as mean ± standard deviation (SD). Intergroup differences were evaluated by one-way ANOVA, and values of *P* < 0.05 were regarded as statistically significant. All assays were carried out in at least three independent replicates.

## 3 Results

### 3.1 Molecular weight determination and thermal analysis of AHPN

The calibration curve for dextran standards was established as lgMw = 0.1306t^2^ – 3.6595t + 29.44 (*R*^2^ = 0.9281). AHPN showed a single symmetric peak at 12.884 min (Figure 1A), corresponding to a molecular weight of 9.35 × 10^3^ Da. The TG, DTG, and DSC curves of AHPN (Figure 1B) revealed a three-step degradation. The first weight loss (27°C and 133°C, rate 10.64%/min) was attributed to loss of free or bound water. The second stage (241.3–360°C) likely corresponded to polysaccharide decomposition and residue transformation, indicating a complex polymer structure.

### 3.2 Scanning electron microscopy of AHPN

As shown in Figure 1C, SEM analysis revealed the microstructure of lyophilized AHPN. At × 500 magnification, an irregular lamellar structure with numerous surface pores was observed. When magnification was increased to × 3000, the lamellae exhibited a smoother surface and contained multiple pores of varying sizes.

### 3.3 FT-IR spectroscopic investigation of AHPN

The FT-IR spectrum of AHPN, recorded across the wavenumber range of 4,000–400 cm^−1^, is displayed in Figure 1D. The broad O–H band at 3,325 cm^−1^ indicates abundant hydroxyls in the polysaccharide. The absorption at 2,932 cm^−1^ arose from C–H stretching vibrations, while the peak at 1,613 cm^−1^ was associated with hydrated water. The band near 1,405 cm^−1^ corresponded to C–H bending. A strong absorption at approximately 1,046 cm^−1^ was assigned to C–O stretching of glycosidic bonds or the pyranose ring. Additionally, the band at around 585 cm^−1^ suggested the presence of α-pyranose configurations.

### 3.4 Chemical analysis of AHPN

Monosaccharide composition analysis revealed that AHPN was mainly comprised of mannose, glucose, and galactose, with relative molar percentages of 29.384, 41.804, and 28.810%, respectively. Glucose constituted the major monosaccharide fraction (Figure 1E).

### 3.5 Effects of AHPN on CCl_4_-induced LF model mice

**FIGURE 2:**
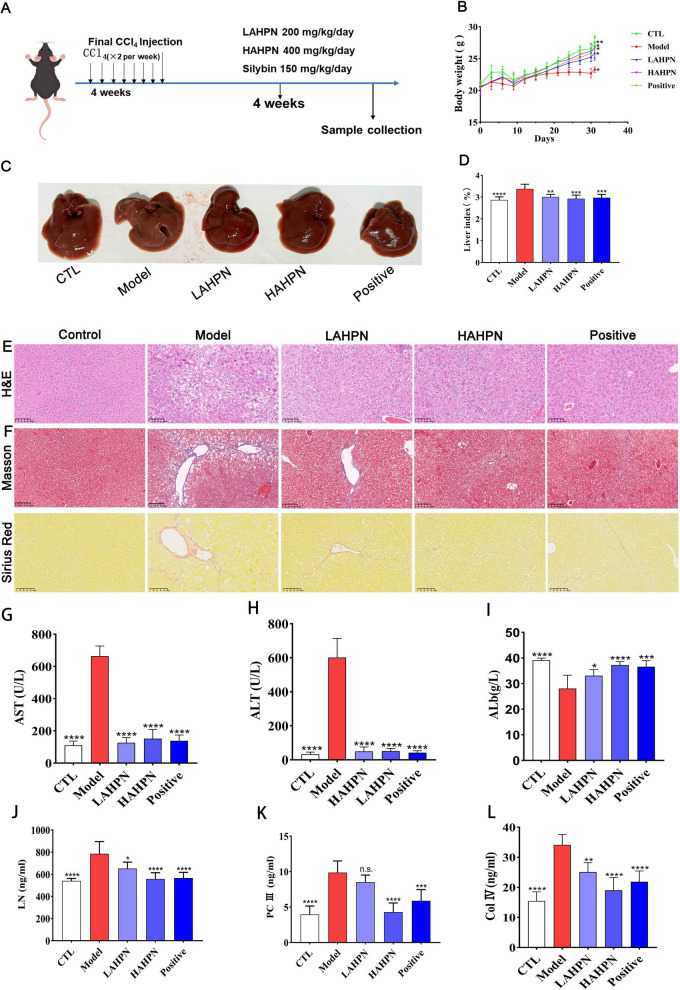
AHPN administration exerts a protective effect against CCl_4_-induced liver injury. **(A)** Liver Fibrosis Model construction and pharmacological treatment procedures. **(B)** Body weight of 5 groups. **(C)** Morphological effects of AHPN on the appearance of liver of rats with liver fibrosis. **(D)** Liver index of five groups. **(E)** HE pathological histology (200×). **(F)** Masson staining (×200), Sirius red staining (×200). **(G–I)** Serum AST, ALT, and ALb activities in mice. **(J)** Liver LN concentration. **(K)** Liver PC III level. **(L)** Liver Col IV concentration (*n* = 5–6). Statistical significance compared to the Model group is indicated as follows: *P < 0.05, **P < 0.01, ***P < 0.001, ****P < 0.0001.

**FIGURE 3:**
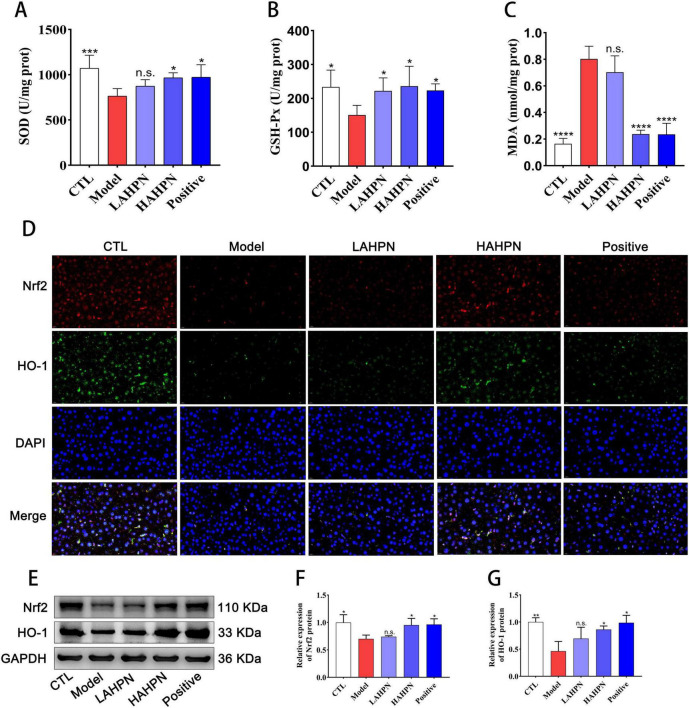
AHPN ameliorated model-induced oxidative stress. **(A–C)** SOD, GSH-Px enzyme activities and liver MDA content. **(D)** Visualization of Nrf2 and HO-1 Co-Expression via Immunofluorescence Staining. **(E)** Representative Western blot images of Nrf2 and HO-1. **(F,G)** Nrf2 and HO-1 protein expression. Results are expressed as mean ± SD (*n* = 3). One-way ANOVA; *p < 0.05, **p < 0.01, ***p < 0.001, ****p < 0.0001 vs. Model group.

Following 4 weeks of intervention (as illustrated in Figure 2A), therapeutic outcomes were assessed through multiple parameters, including body weight, liver index, serum levels of ALT and AST, as well as liver histopathological changes. As presented in Figure 2B, mice in the Model group exhibited a significant reduction in body weight relative to the CTL group (*p* < 0.01), whereas treatment with AHPN markedly alleviated this decline (*p* < 0.01). Macroscopic examination revealed that, compared to controls, livers from Model group animals were enlarged, firm in texture, darker in coloration, and featured diffuse surface nodularity. By contrast, AHPN administration substantially attenuated these CCl_4_-induced morphological alterations (Figure 2C). Moreover, CCl_4_ exposure led to a pronounced elevation in both liver index and serum transaminase activities relative to the CTL group, while AHPN treatment effectively counteracted these biochemical disturbances. AHPN treatment reversed the liver fibrosis index and reduced the activities of AST and ALT in mice with liver fibrosis (Figures 2D, 3A,B). Elevated serum transaminases reflect hepatocellular membrane integrity disruption, while increased LN, PCIII, and Col IV indicate active ECM synthesis—together these parameters characterize the severity of CCl_4_-induced hepatic injury and fibrogenesis. Liver pathological changes after AHPN treatment were observed using H&E, Masson, and Sirius Red staining, as well as immunohistochemical staining. H&E staining (Figure 2E) demonstrated that in the CTL group, hepatocytes were radially oriented, with well-preserved liver architecture. Conversely, histopathological analysis of the Model group revealed disrupted liver cords, hepatocellular swelling and necrosis, nuclear pyknosis or absence, inflammatory infiltration, and fibrotic changes. Treatment with AHPN significantly ameliorated these morphological alterations in the liver tissue. Masson’s trichrome staining (Figure 2F) further demonstrated increased collagen deposition and a higher number of fibrotic foci in the Model group. Sirius Red staining indicated a marked increase in type I collagen in the Model group. After AHPN treatment, collagen deposition and fibrotic lesions were significantly reduced.

Serum biochemical parameters associated with liver injury were assessed to evaluate the impact of AHPN on CCl_4_-induced damage. Compared to control levels, persistent CCl_4_ exposure in Model mice resulted in pronounced elevations of AST, ALT, LN, PCIII, and Col IV, alongside a concomitant decline in ALb concentrations (Figures 2G–L). In contrast, AHPN intervention effectively alleviated the increases in AST, ALT, LN, PC III, and Col IV as well as the decrease in ALb, with the HAHPN (400 mg/kg BW) group showing a more pronounced effect.

### 3.6 AHPN suppresses inflammation and oxidative stress in mice with LF

Liver oxidative status following AHPN treatment was assessed by examining the levels of GSH-Px, SOD, and MDA in liver homogenates. Compared with the CTL group, the Model group displayed a pronounced decrease in both SOD and GSH-Px activities, alongside a significant increase in MDA content. This effect was reversed following AHPN intervention (as shown in Figures 3A–C).

Both immunofluorescence staining and Western blot assays showed that AHPN treatment increased Nrf2 and HO-1 protein levels (Figures 3D–G). Nrf2 and HO-1 are key regulators of cellular antioxidant responses; their upregulation following AHPN administration was accompanied by enhanced SOD and GSH-Px activities and reduced MDA levels, suggesting that AHPN may alleviate CCl_4_-induced hepatic oxidative damage through modulation of this pathway. Accordingly, measuring downstream antioxidant enzymes revealed that AHPN markedly elevated SOD and GSH-Px activities (Figures 3A,B). These results further confirm that AHPN can suppress oxidative damage in mice with liver fibrosis.

### 3.7 AHPN suppresses liver fibrosis-associated protein expression in CCl_4_-induced mice

**FIGURE 4:**
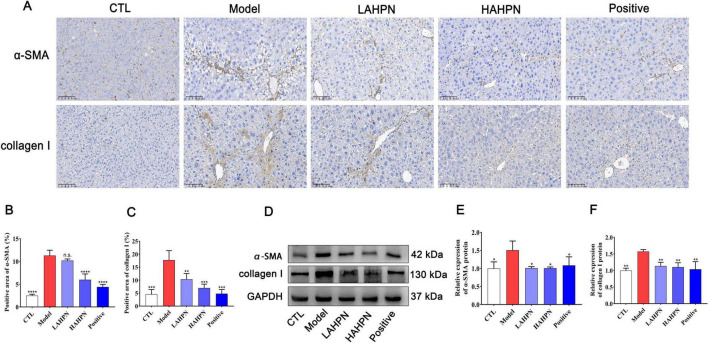
AHPN attenuates liver α-SMA and Collagen I expression in CCl_4_-induced liver fibrotic mice. **(A)** α-SMA and collagen I immunostaining by immunohistochemistry. **(B,C)** The positive rate of immunohistochemical staining for α-SMA and collagen I. **(D)** Western blot detection of α-SMA and Collagen I protein expression. **(E,F)** Quantification of α-SMA and Collagen I Expression (*n* = 3). Significance: *P < 0.05, **P < 0.01, ***P < 0.001, ****P < 0.0001 vs. Model group.

α-SMA is a biomarker of activated hepatic stellate cells (HSCs), while type I collagen is a major component of the ECM (21–23). Therefore, the expression of α-SMA and type I collagen was further examined by immunohistochemistry and Western blot. As shown in Figures 4A–F, the results from immunohistochemistry and Western blot analysis demonstrated that long-term injection of CCl_4_ significantly upregulated the expression of α-SMA and type I collagen in mouse liver tissues compared to the CTL group (*P* < 0.001), indicating that CCl_4_ induced HSC activation and ECM formation. However, AHPN pretreatment significantly downregulated these two proteins, particularly in the HAHPN group. These results indicate that AHPN treatment is associated with attenuation of hepatic stellate cell activation and ECM accumulation, consistent with an antifibrotic effect comparable to that observed in the silybin-treated group.

**FIGURE 5:**
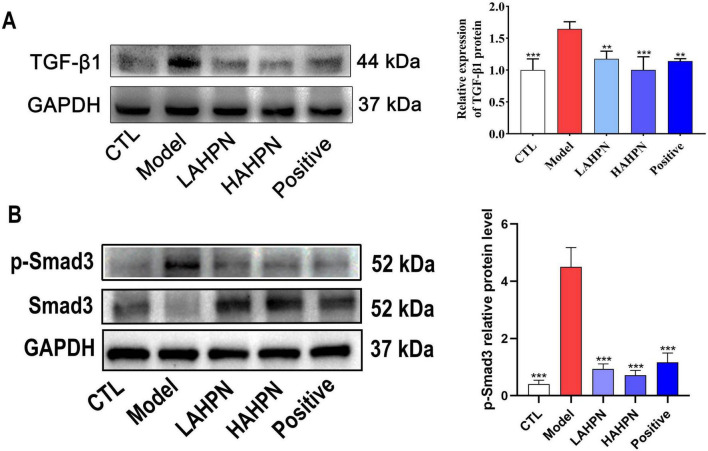
AHPN attenuates TGF-β1/Smad3 signaling pathway in liver fibrosis mouse model. **(A)** Western blot analysis and quantification of TGF-β1 protein expression. **(B)** Western blot analysis and quantification of phosphorylated Smad3 (p-Smad3) and total Smad3 protein levels. GAPDH served as loading control. Data are presented as mean ± SD. **P < 0.01, ***P < 0.001 vs. Model.

Consistent with previous reports linking TGF-β1/Smad3 signaling to liver fibrosis pathogenesis, hepatic TGF-β1 protein levels and Smad3 phosphorylation were evaluated across all experimental groups using Western blot analysis (24, 25). As shown in Figure 5A, prolonged CCl_4_ challenge led to a marked upregulation of TGF-β1 expression in liver tissues compared to the CTL group (*P* < 0.001), confirming that CCl_4_ exposure effectively promoted fibrogenesis, and this pro-fibrotic effect was substantially attenuated following both LAHPN and HAHPN administration in a dose-dependent manner, with efficacy comparable to the Positive control group (*P* < 0.01). Similarly, as shown in Figure 5B, p-Smad3 levels were dramatically elevated in the Model group compared to the CTL group (*P* < 0.001), while total Smad3 protein remained unchanged across all groups, indicating specific activation of the signaling pathway; importantly, AHPN treatment dose-dependently suppressed p-Smad3 activation, with both LAHPN and HAHPN showing significant reductions relative to the Model group (*P* < 0.001), demonstrating that AHPN exerts anti-fibrotic effects by downregulating TGF-β1 expression and subsequently inhibiting downstream Smad3 phosphorylation, thereby blocking canonical TGF-β1/Smad3 signaling in liver fibrosis.

### 3.8 AHPN ameliorates intestinal barrier dysfunction linking gut microbiota and liver fibrosis

**FIGURE 6:**
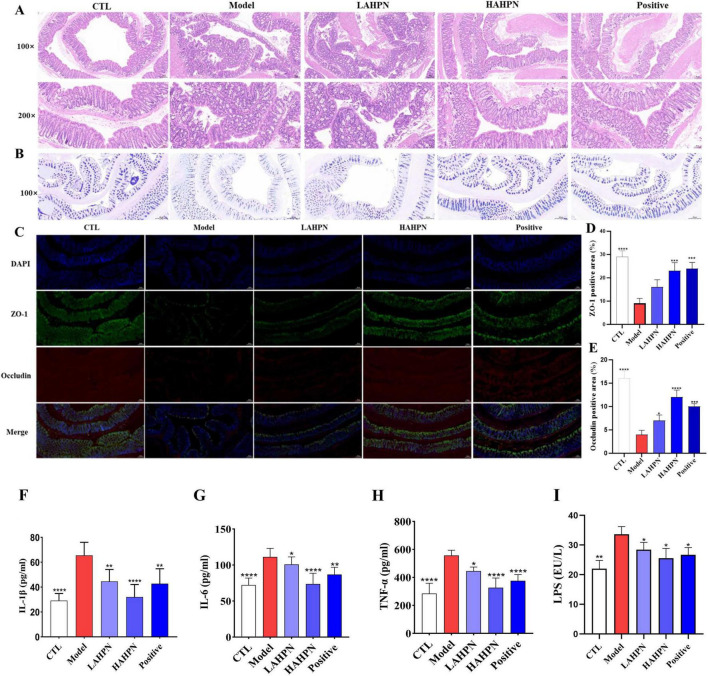
AHPN mitigates intestinal barrier dysfunction in mice. **(A,B)** Histological evaluation via H&E and AB-PAS Staining (100× and 200×). **(C)** Immunofluorescent localization of Occludin and ZO-1. **(D,E)** The positive area ratios of Occludin and ZO-1. **(F–I)** Serum concentrations of IL-1β, IL-6, TNF-α and LPS. Group comparisons were performed by one-way ANOVA, with significance levels set at *P < 0.05, **P < 0.01, ***P < 0.001, and ****P < 0.0001 relative to the Model group.

In the CCl_4_-induced mouse intestinal injury model, histopathological and AB-PAS staining revealed damage to the intestinal villus structure, reduced villus height, decreased glycocalyx and goblet cells, accompanied by inflammatory cell infiltration and epithelial sloughing (Figures 6A,B). Following AHPN intervention, these intestinal pathological changes were significantly ameliorated, and intestinal permeability was restored. HAHPN attenuates CCl_4_-induced intestinal mucosal injury to levels comparable to CTL. The influence of AHPN on intestinal barrier function was further investigated by analyzing the expression levels of tight junction markers occludin and ZO-1 (26). Both proteins were downregulated in the Model group but increased following AHPN intervention, mirroring the morphological improvements, as shown by immunofluorescence (Figures 6C–E). Further assessment of the anti-inflammatory effect of AHPN. Compared to the Control group, the Model group showed increased serum levels of the pro-inflammatory cytokines IL-6, IL-1β, and TNF-α, as well as elevated endotoxin LPS (Figures 6F–I). Following AHPN treatment, the levels of these inflammatory mediators were markedly reduced, with the HAHPN group demonstrating stronger anti-inflammatory effects. Collectively, these findings suggest that AHPN may protect against CCl_4_-induced hepatotoxicity by maintaining intestinal barrier integrity and limiting endotoxin LPS translocation into the portal circulation, which could contribute to reduced hepatic inflammatory signaling.

### 3.9 AHPN reshapes gut microbiota composition to modulate the gut-liver metabolic axis

**FIGURE 7:**
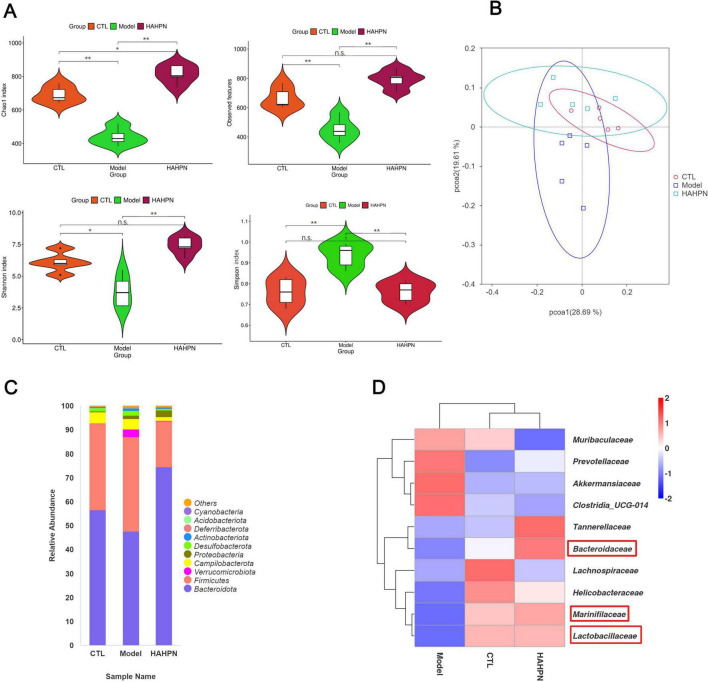
AHPN reversed CCl_4_-induced intestinal dysbiosis. **(A)** Displays four alpha diversity measures—Chao1 index, observed features, Shannon index, and Simpson index—reflecting microbial community richness and evenness. **(B)** presents principal coordinate analysis (PCoA) plots generated from weighted and unweighted UniFrac distances, calculated based on amplicon sequence variant (ASV) profiles. **(C)** Bar plot of bacterial composition at the phylum level. **(D)** Heatmap of bacterial composition at the family level. For assessing between-group variations in alpha diversity measures, the Wilcoxon rank-sum test was applied; statistical significance was defined as *p < 0.05 and **p < 0.01.

Dietary polysaccharides are generally resistant to enzymatic hydrolysis in the upper gastrointestinal tract and reach the colon intact, where they serve as selective substrates for specific gut microbial taxa, thereby reshaping microbial community structure and function. To elucidate the mechanistic basis underlying the anti-fibrotic effects of AHPN, 16S rDNA amplicon sequencing was performed on fecal samples collected from 15 mice to profile alterations in gut microbial community structure. Following PCR amplification of the 16S rDNA region, high-throughput sequencing was conducted to assess alpha diversity, which reflects species richness within each sample. Four complementary metrics were employed: Chao1, observed species, Shannon, and Simpson indices. Chao1, observed species, and Shannon values increase with species richness, whereas the Simpson index decreases. Comparative analysis revealed that CCl_4_ exposure markedly reduced microbial alpha diversity relative to control levels, while subsequent intervention with AHPN significantly restored these parameters (Figure 7A). In principal coordinates analysis (PCoA), differences between samples are represented by the distance between coordinates: a shorter distance indicates higher similarity, while a greater distance indicates lower similarity. PCoA analysis revealed clear separation among the CTL, Model, and HAHPN groups, and differences in gut microbiota composition were identified (Figure 7B). At the phylum level, CCl_4_ exposure induced marked shifts in microbial composition relative to the CTL group, characterized by reduced abundance of *Bacteroidota* alongside enrichment of Firmicutes and *Verrucomicrobiota* (Figure 7C). Following AHPN treatment, this profile was substantially remodeled: *Bacteroidota* and *Proteobacteria* levels increased, whereas *Firmicutes* and *Verrucomicrobiota* declined compared to Model mice. Further taxonomic profiling at the genus level revealed that CCl_4_ administration decreased the relative proportions of *Lactobacillaceae* and *Marinifilaceae* within the intestinal microbiota (Figure 7D). Notably, this depletion was effectively counteracted by AHPN intervention. Together, these results demonstrate that AHPN exerts a modulatory effect on the gut microbial ecosystem, promoting the expansion of potentially beneficial bacterial populations in mice following CCl_4_ challenge.

### 3.10 Validation of the causal relationship between gut microbiota and LF

**FIGURE 8:**
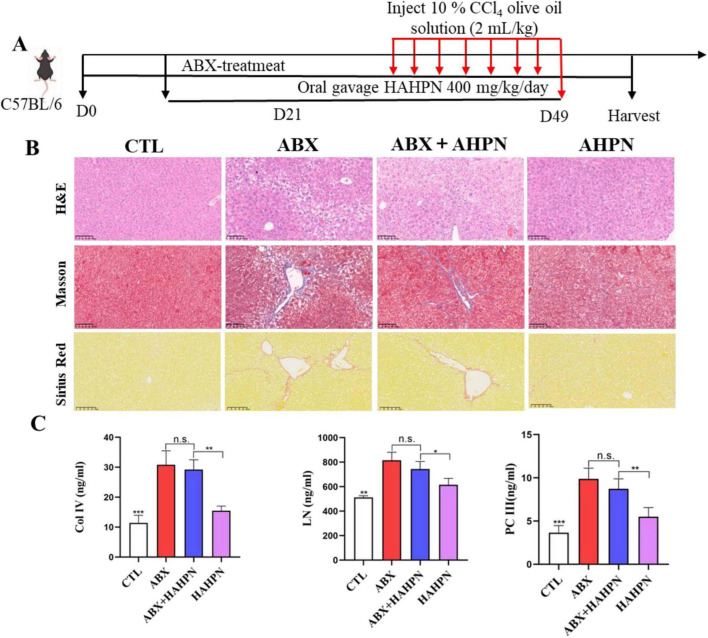
Antibiotic treatment attenuates the effect of AHPN on LF. **(A)** Overall flowchart of the mouse experiment. **(B)** H&E, Masson, and Sirius red staining results in each group. **(C)** Detection results of fibrosis markers including Col IV, LN, and PC III in each group (*n* = 6, *P < 0.05, **P < 0.01, ***p < 0.001).

To further investigate the causal role of gut microbiota in hepatic fibrosis, all mice except the CTL group received a 2-week antibiotic pretreatment to deplete gut microbiota prior to modeling (27). The subsequent experimental protocol is shown in Figure 8A. Histological analyses (H&E, Masson’s trichrome, and Sirius red staining) revealed that, compared with the CTL group, the ABX and ABX + HAHPN groups exhibited marked hepatic disorganization, hepatocyte necrosis, inflammatory infiltration, and fibrosis. In contrast, these pathological changes were ameliorated in the HAHPN group. Masson’s trichrome and Sirius red staining further showed increased collagen deposition and type I collagen in model groups, which were significantly reduced after HAHPN treatment (Figure 8B). Notably, in microbiota-depleted mice (ABX + HAHPN group), HAHPN treatment showed only modest or non-significant reductions in LN, PCIII, and Col IV levels compared with the ABX group (*P* > 0.05). In contrast, HAHPN treatment effectively decreased these fibrotic markers in mice with intact gut microbiota, indicating that the gut microbiota plays an essential role in mediating the anti-fibrotic effects of AHPN (Figure 8C). These findings suggest that the gut microbiota plays a critical role in the protective effects of HAHPN, which exerts anti-fibrotic activity by modulating the gut microbiota.

### 3.11 AHPN remodels hepatic metabolism via the gut microbiota-liver metabolism axis

**FIGURE 9:**
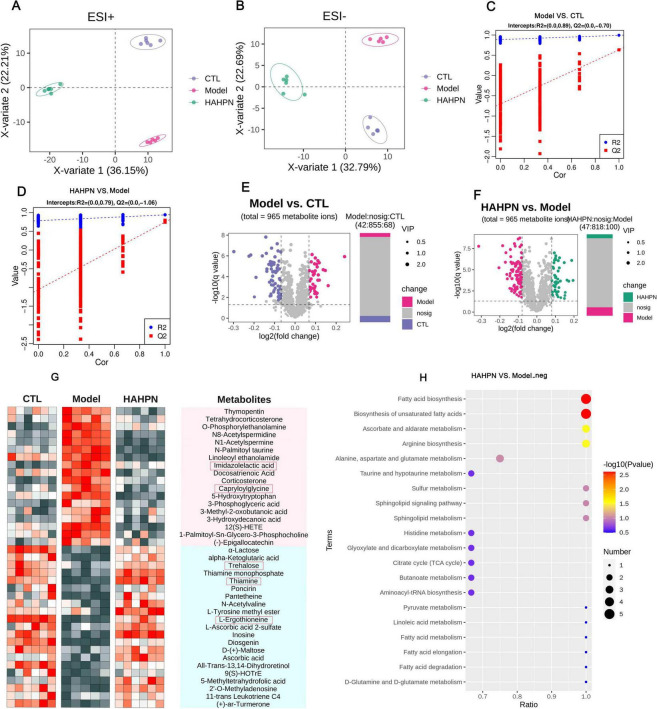
Metabolomic analysis of HAHPN in liver fibrosis mice. **(A,B)** PCA plots of liver metabolomics in ESI + and ESI- modes. **(C,D)** PLS-DA permutation validation (*n* = 200, ESI + /ESI- ). **(E,F)** Volcano plots of metabolites in the CTL, Model, and HAHPN groups under ESI + and ESI- modes, respectively. **(G)** Hierarchical clustering analysis heatmap of partial differential metabolites between the Model and HAHPN groups. **(H)** KEGG pathway analysis of differential metabolites between the Model and HAHPN groups.

To untangle the impact of HAHPN administration on metabolic phenotypes and identify subtle inter-group differences, a multivariate statistical analysis strategy was employed, including unsupervised Principal Component Analysis (PCA) and supervised Partial Least Squares Discriminant Analysis (PLS-DA). The PCA score plots (Figures 9A,B), constructed based on peak alignment data from positive and negative ion modes, clearly demonstrated significant separation in the metabolic profiles among the groups. Furthermore, the HAHPN group and the Model group exhibited distinct separation in the PLS-DA score plots (Figures 9C,D), indicating that HAHPN intervention effectively reversed the CCl_4_-induced hepatic metabolic disorders. Validated by 200 permutation tests, the PLS-DA model demonstrated robust discriminative ability and predictive performance in both ion modes, excluding overfitting and confirming its reliability for subsequent biomarker screening. Based on the PLS-DA model, differential metabolites were identified using a Variable Importance in Projection (VIP) threshold of ≥ 1.0. Their distribution was visualized via volcano plots (Figures 9E,F). Figure 9G (heatmap) showed clear metabolite profile differences between HAHPN and Model groups. Collectively, these metabolic profile alterations suggest that HAHPN may alleviate CCl_4_-induced liver fibrosis by modulating hepatic metabolic processes. To identify metabolic pathways modulated by HAHPN intervention, enrichment analysis was performed on the identified differential metabolites. As illustrated in the KEGG bubble chart (Figure 9H), several pathways were significantly enriched, including fatty acid biosynthesis, arginine biosynthesis, alanine, aspartate and glutamate metabolism, taurine metabolism, pyruvate metabolism, and butanoate metabolism, suggesting the involvement of these metabolic routes in the protective effects of AHPN.

**FIGURE 10:**
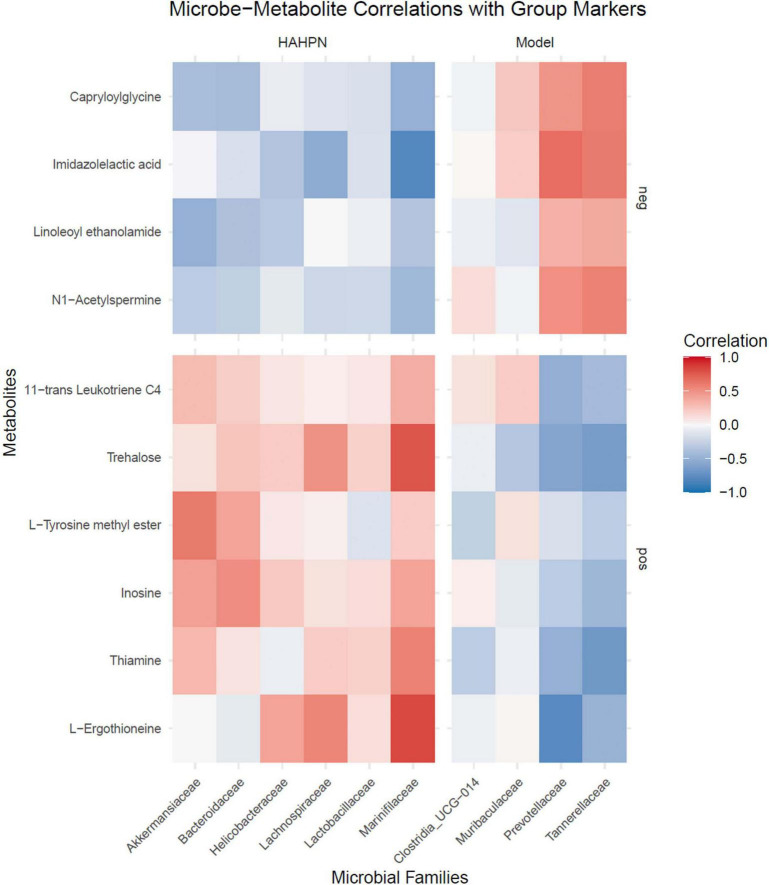
Correlation analysis of microbiota and metabolites. Only correlations with |ρ| > 0.5 and raw two-tailed *P* < 0.05 are shown. Positive correlations are red, negative correlations blue. Color intensity is proportional to |ρ|.

Correlation analysis between microbiota and metabolites revealed markedly different correlation patterns in the HAHPN group compared with the Model group (Figure 10). In the HAHPN group, marked alterations were noted in the correlations of several key metabolites—including Capryloylglycine, Imidazolelactic acid, Linoleoyl ethanolamide, and N1–Acetylspermine—with specific microbiota (all *p* < 0.05), suggesting that AHPN may influence host metabolism by modulating the structure of the intestinal flora. Among these, Capryloylglycine, an intermediate product of fatty acid metabolism, may reflect improved mitochondrial β-oxidation function. As a product of histidine metabolism, imidazolelactic acid may play a regulatory role in inflammation and redox homeostasis. Linoleoyl ethanolamide, an endocannabinoid-like substance, exhibits anti-inflammatory and energy metabolism regulatory effects. N1–Acetylspermine is related to polyamine metabolism and may influence cell proliferation and hepatic repair processes. Taken together, these results demonstrate that HAHPN achieves its anti-fibrotic effects through modulation of the gut microbial community. The resulting shifts in the microbiota–metabolite network modulate host metabolic pathways—such as fatty acid, inflammatory, and polyamine pathways—thereby helping prevent and treat liver fibrosis.

## 4 Discussion

Liver fibrosis is a wound-healing response to chronic parenchymal injury and a major driver of cirrhosis and liver-related mortality. Despite its reversible nature in early stages, no anti-fibrotic therapy has achieved clinical approval, largely due to incomplete mechanistic understanding and inadequate target engagement (28–30). The present study demonstrates that AHPN, as a dietary polysaccharide, attenuates CCl_4_-induced hepatic fibrosis through coordinated modulation of the gut microbiota-liver metabolism axis, integrating intestinal barrier restoration, microbial community reshaping, and hepatic metabolic reprogramming.

The protective effects of AHPN were evidenced by dose-dependent reductions in serum transaminases, collagen deposition, and fibrosis markers (LN, PCIII, Col IV). These histological and biochemical improvements were associated with downregulation of TGF-β1/Smad3 signaling and reduced hepatic stellate cell activation, as indicated by decreased α-SMA and collagen I expression. While these observations are consistent with an inhibitory effect on this pathway, they represent correlational evidence that requires validation through targeted intervention studies to establish causality. TGF-β1 serves as the principal profibrogenic cytokine, driving Smad3-dependent transcription of extracellular matrix genes (25). Our finding that AHPN downregulates hepatic TGF-β1 protein levels and Smad3 phosphorylation—while leaving total Smad3 unaffected—indicates specific pathway inhibition rather than generalized protein suppression. This positions TGF-β1/Smad3 as a critical intracellular target, though the upstream regulators remained to be elucidated. Given the established role of oxidative stress as an initiator of TGF-β1 activation and fibrogenesis, we examined the Nrf2/HO-1 antioxidant axis (31). CCl_4_-generated free radicals typically deplete glutathione reserves and propagate lipid peroxidation, creating a permissive environment for fibrotic progression (11). AHPN treatment was associated with increased Nrf2 and HO-1 protein levels, restored SOD and GSH-Px activities, and reduced MDA accumulation. These changes suggest activation of the Nrf2/HO-1 antioxidant axis, which may contribute to the observed reduction in TGF-β1 expression, although direct causal evidence linking Nrf2 activation to TGF-β1 suppression in this model requires further investigation (32). However, antioxidant modulation alone cannot fully account for AHPN’s efficacy, particularly given the magnitude of histological improvement relative to oxidative stress markers.

The intestinal dimension emerged as a critical node when AHPN substantially restored tight junction integrity (Occludin, ZO-1) and reduced systemic LPS translocation. The gut-liver axis provides the anatomical and physiological basis for this interaction: intestinal barrier dysfunction permits bacterial endotoxins and metabolites to access the portal circulation, thereby activating hepatic Toll-like receptors and profibrogenic signaling (33–35). In our model, CCl_4_ induced characteristic villus atrophy and glycocalyx depletion, paralleling the hepatic injury. AHPN reversed these morphological alterations and suppressed serum IL-6, IL-1β, and TNF-α—cytokines whose hepatic expression is strongly LPS-dependent. The functional necessity of an intact microbiota was demonstrated through antibiotic depletion experiments, wherein AHPN lost its protective efficacy against fibrosis when the microbial community was eradicated. While this establishes that gut microbiota are required for AHPN’s effects, it does not distinguish between direct microbial targeting by AHPN and microbial biotransformation of AHPN into bioactive metabolites. Future studies employing isotope-labeled polysaccharide tracing and portal vein metabolomics are needed to resolve this ambiguity. 16S rDNA sequencing revealed that AHPN enriched *Bacteroidota* and specific beneficial taxa including *Lactobacillaceae* and *Marinifilaceae*, while reducing *Firmicutes* and *Verrucomicrobiota* expansion characteristic of CCl_4_-induced dysbiosis. These compositional shifts correlated with restored alpha diversity and distinct beta-diversity clustering, indicating ecosystem-level stabilization rather than single-species enrichment (36, 37). The *Marinifilaceae* genera identified here is less commonly reported in antifibrotic polysaccharide studies compared to canonical probiotics such as *Lactobacillus* or *Bifidobacterium*, suggesting AHPN may engage distinct microbial metabolic pathways. Untargeted hepatic metabolomics provided the mechanistic link between intestinal microbial alterations and liver phenotype. AHPN reversed CCl_4_-induced disturbances in glycerophospholipid, glycine, taurine, and fatty acid metabolism—pathways intimately connected to mitochondrial function, membrane integrity, and redox homeostasis. Notably, taurine upregulation may reinforce antioxidant defenses through its established roles in bile acid conjugation and membrane stabilization (38). Correlation analysis further identified capryloylglycine, imidazolelactic acid, and linoleoyl ethanolamide as metabolites significantly associated with AHPN-responsive microbial taxa. Capryloylglycine reflects enhanced mitochondrial β-oxidation capacity; imidazolelactic acid, derived from histidine metabolism, may modulate inflammatory and redox responses; linoleoyl ethanolamide possesses documented anti-inflammatory properties through endocannabinoid-related pathways. These microbe-metabolite associations suggest that AHPN-induced microbial remodeling generates a distinct metabolic signature that propagates from intestine to liver via the portal circulation.

**FIGURE 11:**
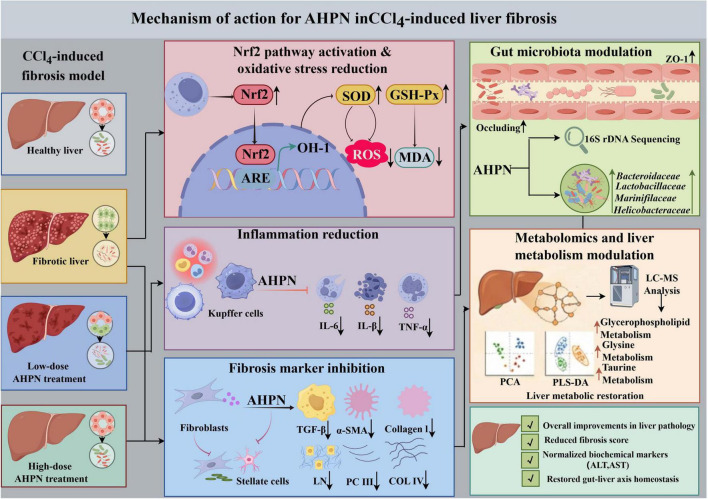
Schematic illustration of AHPN ameliorating liver fibrosis via the gut microbiota-liver metabolism axis.

Synthesizing these observations, we propose that AHPN operates through a hierarchical, axis-centered mechanism (Figure 11). At the intestinal level, AHPN restores epithelial barrier architecture and tight junction protein expression, thereby limiting LPS and other bacterial products from reaching the liver. Concurrently, microbial community restructuring—particularly enrichment of *Bacteroidota* and beneficial families—generates bioactive metabolites that enter portal circulation and reprogram hepatic metabolic pathways. These metabolic alterations, encompassing enhanced fatty acid oxidation, taurine-mediated antioxidant support, and improved mitochondrial function, collectively suppress oxidative stress and inflammatory signaling. The resulting reduction in hepatic injury attenuates TGF-β1 release from damaged hepatocytes and activated Kupffer cells, thereby interrupting Smad3-mediated stellate cell activation and collagen deposition. However, it should be noted that direct evidence for intestinal metabolite transfer to the liver via portal circulation was not obtained in this study; future experiments involving portal vein metabolomics or labeled polysaccharide tracing are warranted to validate this proposed mechanism. This model positions the gut microbiota-liver metabolism axis as the primary regulatory hub, with antioxidant and anti-inflammatory effects serving as downstream consequences.

This study carries implications for the nutritional application of polysaccharide-based interventions. Unlike single-target pharmaceutical agents that have consistently faced challenges in clinical translation, AHPN engages multiple coordinated pathways through a physiological axis central to metabolic homeostasis, highlighting the advantage of dietary bioactive compounds in systems-level modulation. The traditional application of Alhagi honey in hepatobiliary disorders within Uyghur medicine finds mechanistic validation in our demonstration of gut-liver axis modulation, bridging ethnomedicinal knowledge with modern systems biology. Future investigations should employ fecal microbiota transplantation to establish microbial causality definitively, isolate specific AHPN-responsive bacterial strains for functional characterization, and trace labeled polysaccharide metabolites to confirm intestinal-to-hepatic metabolite transfer.

## 5 Conclusion

AHPN protects the liver by modulating the gut microbiota-liver metabolism axis. In the gut, it reshapes microbiota composition, boosts beneficial bacteria, repairs barrier function, and limits endotoxin entry into the liver. Metabolically, it corrects liver disorders, strengthens antioxidant defenses, optimizes energy balance, and activates anti-fibrotic signals. This bidirectional axis explains AHPN’s multi-target effects.

## Data Availability

The original contributions presented in this study are included in the article/supplementary material, further inquiries can be directed to the corresponding authors.
